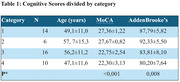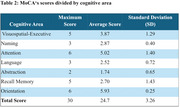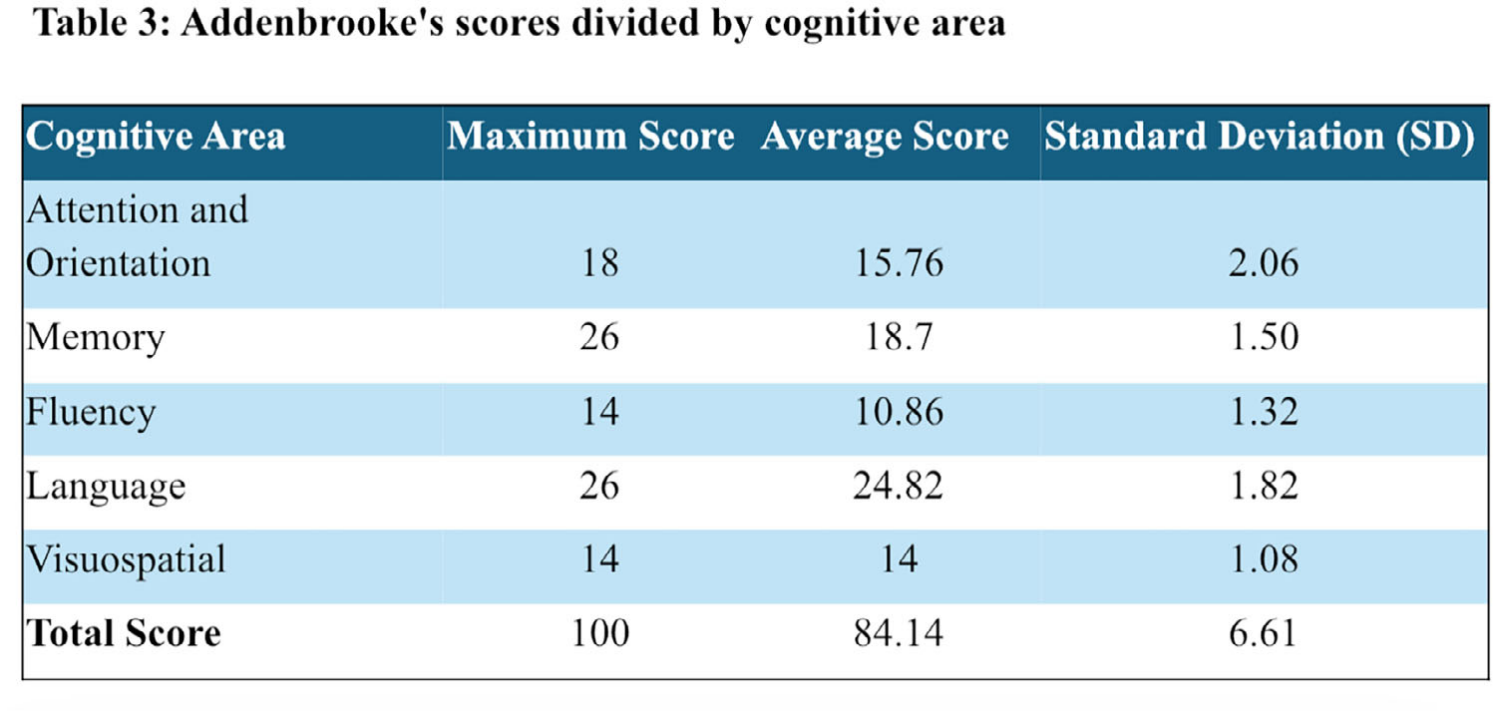# Profiling cognitive impairment in Post COVID Syndrome

**DOI:** 10.1002/alz70860_107241

**Published:** 2025-12-23

**Authors:** Leidys Pedrozo Garcia, Eduarda Rosa Fernandes, Carolina Heck Haubrich, Andrei Bieger, William Alves Martins, Jaderson Costa da Costa

**Affiliations:** ^1^ São Lucas Hospital, Pontifícia Universidade Católica do Rio Grande do Sul (HSL‐PUCRS), Porto Alegre, Rio Grande do Sul, Brazil; ^2^ São Lucas Hospital, Pontifícia Universidade Católica do Rio Grande do Sul, Porto Alegre, Rio grande do sul, Brazil; ^3^ Neurology Department, São Lucas Hospital of PUCRS, Porto Alegre, Rio Grande do Sul, Brazil; ^4^ Instituto do Cérebro do Rio Grande do Sul, Porto Alegre, RS, Brazil

## Abstract

**Background:**

Post‐COVID Syndrome is characterized by symptoms persisting for over 12 weeks after acute infection, and may include fatigue, emotional issues, and cognitive impairments. This study aims to investigate the cognitive impacts and identify predisposing factors in individuals affected by this syndrome.

**Method:**

We analyzed 46 patients who survived severe COVID‐19 and were admitted at Hospital São Lucas da PUCRS, Brazil, between January and March 2021. Those were evaluated 2 years and 7 months after the infection. Data collection included questionnaires assessing sociodemographic factors, comorbidities, Hamilton scales for depression and anxiety, and the SF‐36 quality‐of‐life questionnaire. Participants were categorized based on cognitive and physical assessments using the MoCA and Functional Capacity (FC) scores:

Cognitivelly Unimpaired (CU): MoCA ≥26, FC ≥70

Functional Impairment (FC): MoCA ≥26, FC ≤70

Cognitive Impairment (CI): MoCA ≤26, FC ≥70

Cognitive and Functional Impairment (CI‐FC): MoCA ≤26, FC ≤70

**Result:**

Mean age of participants was 52.2 years (SD 12.1), with 45.7% being female and 55% having completed higher education. A total of 45.7% were sedentary and 41.3% were overweight. Regarding comorbidities, 15.2% had diabetes, 45.7% had hypercholesterolemia and 34.8% had systemic arterial hypertension.

Among CU participants, 14.3% reported mild to moderate depression, with minimal cognitive impairment (MoCA: 27.36, ACE‐III: 87.79). FC showed significant functional decline (score: 52.17), but emotional aspects remained intact. In the CI group, 6.3% had moderate depression with noticeable memory decline (MoCA: 22.75, ACE‐III: 83.81). CI‐FC participants, 40% exhibited severe cognitive impairment (MoCA: 22.30, ACE‐III: 80.2).

The average MoCA score for participants was 2.70 (SD 1.48) out of 5, while the average ACE‐III score was 18.7 (SD 1.5) out of 26. Both tests showed alterations in retrograde memory and delayed recall, with a significant *p*‐value < 0.05. (Table 1)

**Conclusion:**

The study highlights persistent cognitive deficits even years after infection (Table 2 and 3). This contrasts with the expected cognitive decline associated with functional impairment in the continuum of normal and pathological aging, as described in the 2022 Brazilian Dementia Consensus.